# Cosmetic-Associated Acute Paronychia With Green Nail-Bed Pigmentation in a Child Following Nail Polish Application

**DOI:** 10.7759/cureus.112633

**Published:** 2026-07-14

**Authors:** Hiroshi Fukumasa, Komei Asabe, Yukihiro Tasaki

**Affiliations:** 1 Department of Emergency Medicine, Kitakyushu City Yahata Hospital, Kitakyushu, JPN; 2 Department of Plastic Surgery, Hamanomachi Hospital, Fukuoka, JPN; 3 Department of Plastic Surgery, Kitakyushu City Yahata Hospital, Kitakyushu, JPN

**Keywords:** acute paronychia, children, cosmetic-related infection, nail cosmetics, pediatric nail disorders

## Abstract

Acute paronychia in children is typically associated with trauma or habitual behaviors, whereas cosmetic-related cases are rarely reported. We describe a 4-year-old girl who developed acute paronychia with persistent green nail-bed pigmentation following the application of nail polish to a nail with pre-existing cuticular disruption. *Staphylococcus epidermidis* was isolated from purulent discharge, while *Pseudomonas aeruginosa* was not detected. The clinical course supports a dual pathogenic mechanism involving bacterial invasion through a compromised cuticle and chemical irritation from cosmetic components. The observed green discoloration, confined to the nail bed and occurring in the absence of *P. aeruginosa*, represents an atypical presentation distinct from classical chloronychia. This case highlights that cosmetic products may represent an underrecognized trigger of nail infections in children, particularly when applied to compromised nail units, and underscores the need for clinician awareness as the use of children’s cosmetic products continues to expand.

## Introduction

Acute paronychia is one of the most common infections involving the nail apparatus in children. It typically develops following disruption of the protective nail fold or cuticular barrier, allowing microorganisms to invade the periungual tissues. Common predisposing factors include minor trauma, nail biting, thumb sucking, and manipulation of the nail unit [1,2].

In a retrospective case series of pediatric patients who underwent surgical drainage for paronychia, *Staphylococcus aureus* was the most frequently isolated pathogen, although a wide variety of microorganisms were identified, highlighting the heterogeneous microbiology of pediatric paronychia [1].

In adults, nail polish has been recognized as a risk factor for acute paronychia [3-6]. The increasing availability and use of cosmetic products among children may introduce additional risk factors for nail disorders. However, cosmetic-associated paronychia in children has rarely been described.

Here, we describe a 4-year-old girl who developed acute paronychia with green nail-bed pigmentation following nail polish application to a previously injured nail. Green nail discoloration is generally associated with *Pseudomonas aeruginosa *infection (chloronychia) [7, 8]. However, *P. aeruginosa *was not isolated in the present case despite the presence of green nail discoloration, representing an atypical clinical presentation. This case highlights a potentially underrecognized cosmetic-associated trigger of nail infection in children.

## Case presentation

A previously healthy 4-year-old girl presented to the emergency department with a 1-day history of pain and swelling localized to the distal aspect of her right thumb. Vital signs were within the normal range. Physical examination revealed erythema, swelling, and tenderness extending from the nail plate to the interphalangeal joint. Distinct green discoloration was observed at the proximal nail fold and cuticle, subtly extending to the nail bed. A linear crack-like lesion was noted in the center of the cuticle, suggesting that the injury primarily involved the cuticle rather than the nail plate (Figure 1A).

According to her mother, she had injured her right thumbnail during a sibling fight the previous day, complaining of a cracked nail, although her mother did not examine it closely. Later that day, the patient independently applied commercially available pink nail polish to the injured nail.

A diagnosis of acute paronychia was made. Needle drainage using an 18-gauge needle yielded bright green purulent discharge (Figure 1B). The purulent material emitted an odor resembling an organic solvent, possibly related to residual nail polish components. The purulent material was submitted for routine aerobic bacterial culture, which identified *Staphylococcus epidermidis*. Because drainage resulted in incomplete resolution with a residual subungual abscess, a plastic surgeon was consulted. Complete nail plate removal was subsequently performed (Figure 1C).

**Figure 1:**
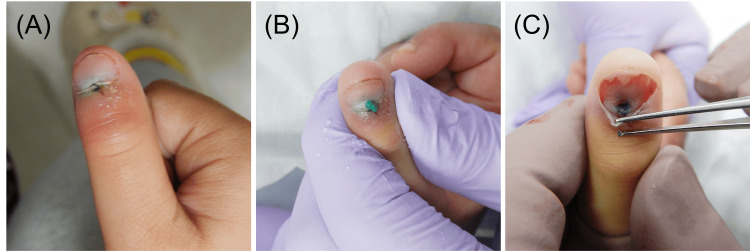
Clinical findings of cosmetic-associated acute paronychia. (A) Erythema, swelling, and tenderness extending from the nail plate to the interphalangeal joint. Green discoloration is observed at the proximal nail fold and cuticle, extending to the nail bed. A linear crack-like lesion is present at the center of the cuticle, indicating disruption of the cuticular barrier. (B) Needle drainage yields bright green purulent discharge. (C) Complete removal of the nail plate reveals a subungual abscess. Residual green pigmentation remains visible in the nail bed after nail plate removal.

The patient was discharged with oral cefaclor and scheduled for outpatient follow-up at our hospital. However, her parents discontinued follow-up at our institution, and she was subsequently evaluated at a nearby dermatology clinic. According to the dermatologist's report, the infection resolved; however, green nail-bed discoloration persisted. No further clinical follow-up information was available thereafter.

## Discussion

To our knowledge, reports of cosmetic-associated acute paronychia in children remain extremely limited. In adults, cosmetic products such as nail polish have been recognized as potential risk factors for acute paronychia [3-6]; however, similar cases have rarely been described in pediatric patients. In the present case, the infection may have been precipitated by the application of nail polish to a nail with pre-existing cuticular disruption, leading to secondary bacterial involvement of the nail bed.

The temporal sequence of nail trauma, immediate nail polish application, and subsequent purulent inflammation suggests a possible dual pathogenic mechanism: (1) bacterial invasion through a compromised cuticle and (2) chemical irritation induced by cosmetic components. *Staphylococcus epidermidis*, isolated from the lesion, is a commensal organism that may act as an opportunistic pathogen in the setting of barrier disruption. McKean et al. reported that *Staphylococcus aureus* was the most frequently identified pathogen in pediatric paronychia, followed by *Staphylococcus spp*. (40%), underscoring the clinical relevance of coagulase-negative staphylococci such as *S. epidermidis* [1]. Although contamination cannot be entirely excluded, the presence of green purulent discharge, localized abscess formation, and clinical improvement after drainage and antimicrobial therapy support a true infectious process.

Management of acute paronychia is primarily determined by the presence of an abscess. Current recommendations emphasize adequate drainage as the cornerstone of treatment, while systemic antibiotics are generally reserved for patients with surrounding cellulitis, systemic illness, immunocompromise, or severe infection [9]. In the present case, a subungual abscess was identified and initially treated with needle drainage. Because drainage alone resulted in incomplete resolution, complete nail plate removal was subsequently performed. Green discoloration remained evident within the exposed nail bed after nail plate removal, suggesting persistent involvement of the underlying periungual tissues. Therefore, empiric oral antibiotic therapy was initiated following surgical drainage. Cefaclor, a second-generation cephalosporin, was selected empirically because the patient had no systemic signs of infection and was later supported by the isolation of *S. epidermidis*, which was considered susceptible.

The green discoloration observed in this case differs from classical *P. aeruginosa*-associated chloronychia, which typically involves the nail plate and produces a characteristic yellow-green hue [7,8]. In contrast, *P. aeruginosa* was not detected, and the pigmentation was confined to the nail bed rather than the nail plate, making classic green nail syndrome unlikely. Although contamination or false-negative culture results cannot be completely excluded, the overall clinical and microbiological findings argue against these possibilities.

The precise mechanism underlying the observed discoloration remains uncertain. One plausible explanation is pigment alteration resulting from interactions between nail polish components and inflammatory exudates or microbial activity. Certain cosmetic pigments have been reported to exhibit limited stability and color variation under environmental conditions such as changes in pH or microbial metabolism [10]. However, this explanation remains speculative because the responsible pigment or chemical compound was not identified in the present case.

The safety of cosmetic use in children has traditionally been discussed in the context of accidental ingestion of nail polish or nail polish removers. In contrast, adverse events resulting from inappropriate topical application have received comparatively little attention. In recent years, the children's cosmetics market has expanded rapidly [11], with an increasing number of products marketed as "safe" and "non-toxic," including water-based formulations free of parabens, phthalates, and formaldehyde. These products are widely available, even in low-cost retail settings, increasing opportunities for routine cosmetic use among children. Consequently, clinicians are likely to encounter an increasing spectrum of cosmetic-related adverse events beyond accidental ingestion. This case highlights cosmetic products as a potentially underrecognized trigger of nail infection, particularly when applied to compromised nail units. Clinicians should therefore remain vigilant for such presentations and provide appropriate guidance regarding the safe use of cosmetic products in pediatric populations.

This report is not intended to discourage the use of children's nail polish but rather to emphasize the importance of safe application practices, particularly avoiding cosmetic use on injured nails or periungual tissues and ensuring appropriate parental supervision. These principles may also apply to other cosmetic products intended for children.

This report has several limitations. The exact chemical composition of the nail polish was unavailable; therefore, the specific cosmetic component potentially involved in the development of infection or nail-bed discoloration could not be identified. In addition, no further long-term follow-up information was available after referral to the dermatology clinic.

## Conclusions

We report a rare case of acute paronychia with green nail-bed pigmentation in a child following nail polish application. The temporal association between cuticular injury and subsequent nail polish use suggests that disruption of the cuticular barrier may have facilitated the development of infection and nail-bed discoloration. This case highlights the importance of avoiding the use of cosmetic products on damaged nails and periungual tissues, particularly in children. As the use of pediatric cosmetic products continues to increase, clinicians should be aware of potential cosmetic-related adverse events involving the nail unit and consider cosmetic exposure in the differential diagnosis of pediatric nail disorders.

## References

[1] McKean AR, Williams GJ, Macneal P, Moore LS, Idowu A, Milroy C (2022). Paediatric paronychia: a single centre retrospective, microbiological analysis and national survey. J Plast Reconstr Aesthet Surg.

[2] Harness N, Blazar PE (2005). Causative microorganisms in surgically treated pediatric hand infections. J Hand Surg Am.

[3] Shafritz AB, Coppage JM (2014). Acute and chronic paronychia of the hand. J Am Acad Orthop Surg.

[4] Relhan V, Bansal A (2022). Acute and chronic paronychia revisited: a narrative review. J Cutan Aesthet Surg.

[5] Tyagi M, Singal A (2023). Nail cosmetics: what a dermatologist should know!. Indian J Dermatol Venereol Leprol.

[6] Desai AD, Wang Y, Nadarajah CC, Lipner SR (2022). Cross-sectional analysis of paronychias in the National Electronic Injury Surveillance System 1999-2018. Skin Appendage Disord.

[7] Baran R, Richert B (2024). Green nail: etiology and treatment of chloronychia. Hand Surg Rehabil.

[8] Chiriac A, Brzezinski P, Foia L, Marincu I (2015). Chloronychia: green nail syndrome caused by Pseudomonas aeruginosa in elderly persons. Clin Interv Aging.

[9] Leggit JC (2017). Acute and chronic paronychia. Am Fam Physician.

[10] Negi A (2025). Natural dyes and pigments: sustainable applications and future scope. Sustain Chem.

[11] TechSci Research (2025). Children's cosmetics market - Global industry size, share, trends, opportunity, and forecast, 2018-2028. https://www.techsciresearch.com/report/children-s-cosmetics-market/28549.html.

